# Addressing annotation and data scarcity when designing machine learning strategies for neurophotonics

**DOI:** 10.1117/1.NPh.10.4.044405

**Published:** 2023-08-24

**Authors:** Catherine Bouchard, Renaud Bernatchez, Flavie Lavoie-Cardinal

**Affiliations:** a CERVO Brain Research Centre, Québec, Québec, Canada; b Université Laval, Institute Intelligence and Data, Québec, Québec, Canada; c Université Laval, Département de psychiatrie et de neurosciences, Québec, Québec, Canada

**Keywords:** machine learning, active learning, weakly supervised learning, domain adaptation, image analysis

## Abstract

Machine learning has revolutionized the way data are processed, allowing information to be extracted in a fraction of the time it would take an expert. In the field of neurophotonics, machine learning approaches are used to automatically detect and classify features of interest in complex images. One of the key challenges in applying machine learning methods to the field of neurophotonics is the scarcity of available data and the complexity associated with labeling them, which can limit the performance of data-driven algorithms. We present an overview of various strategies, such as weakly supervised learning, active learning, and domain adaptation that can be used to address the problem of labeled data scarcity in neurophotonics. We provide a comprehensive overview of the strengths and limitations of each approach and discuss their potential applications to bioimaging datasets. In addition, we highlight how different strategies can be combined to increase model performance on those datasets. The approaches we describe can help to improve the accessibility of machine learning-based analysis with limited number of annotated images for training and can enable researchers to extract more meaningful insights from small datasets.

## 1 Introduction

The recent emergence of machine learning approaches has transformed the landscape of biomedical data analysis. Since the first demonstration that the U-Net could be successfully applied to single cell segmentation on a limited number of training samples,^1^ important efforts have been made in developing machine learning tools that are accessible to the bioimaging community.^2^^–^^5^ Such tools include user interfaces that integrate image visualization, labeling, training, and prediction, such as Ilastik^5^ and Napari,^6^ as well as pre-trained algorithms designed to be easily applied to new data, such as Cellpose^4^ and deepImageJ.^7^ Efforts are also being made to facilitate access to the computer resources needed to train the models for microscopy image analysis, which are often a barrier, such as ZeroCostDL4Mic^2^ and the BioImage Model Zoo.^3^ However, supervised machine learning models require datasets that are specifically processed and annotated for the task that they are designed for (e.g., segmentation, detection, and classification). While models can be pretrained on open-access bioimaging datasets,^3^^,^^8^^–^^10^ a fine-tuning step with a subset of annotated data needs to be performed to adapt the model to a new bioimaging analysis task. The annotated training datasets need to be large enough to represent accurately the full data distribution to ensure that the model generalizes well at inference. When the model is applied to a new dataset or to a new batch of images, changes in the features defining the objects of interest may reduce the performance or require the model to be optimized. For biological experiments, this means that any change in the experimental settings may require re-annotation of a subset of the data and retraining or fine-tuning of the model to fit the new data distribution. In research fields, such as neurophotonics, in which data acquisition is costly and annotation requires trained experts, strategies need to be developed to mitigate annotation complexity and increase the robustness of machine learning models to data variability and data imbalance. To support the democratization of machine learning approaches for biomedical image analysis, sharing of open-source models and open-access datasets needs to be combined with optimized and simple annotation strategies accessible to domain experts. Here, we address two aspects that can potentially increase the accessibility to annotated data in neurophotonics: (1) labeling complexity and (2) data scarcity. We present methods we have applied to facilitate the application of machine learning to solve real neurophotonics-specific challenges we have encountered.

## 2 Discussion

### 2.1 Labeling Complexity

Training fully supervised machine learning models to perform analysis tasks on bioimaging datasets is challenging as it requires large amount of precisely annotated images.^2^^,^^11^^,^^12^ Considering that the acquisition of new data or the annotations of the full dataset is not always possible due to ethical, time, or cost constraints, alternative strategies need to be proposed to democratize machine learning approaches in the field of neurophotonics.

**Fig. 1:**
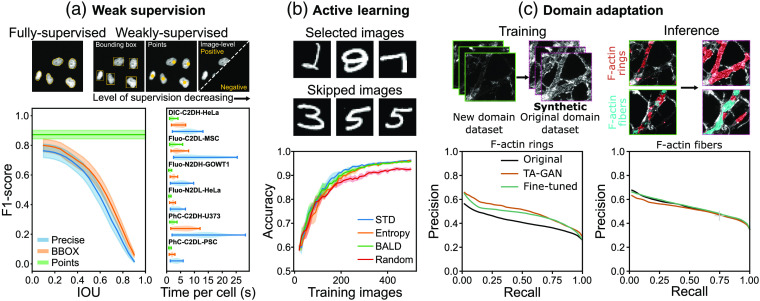
Applications of weak supervision, active learning, and domain adaptation. (a) Similar performance is obtained for a segmentation task on the cell tracking challenge using weakly and fully supervised training schemes. Weak supervision significantly reduces the annotation time.^13^ (b) Active learning allows to obtain a better classification performance with fewer training images compared to random selection of images.^14^ (c) Domain adaptation can help apply a previously trained model to new images from a different domain (e.g., batch, device) by adapting the new images to the original domain.^15^

We have recently addressed the challenge of labeling scarcity using weakly supervised learning approaches. We demonstrated that bounding boxes and binary annotations can replace precise contour annotations to train deep learning models on different tasks (instance segmentation, semantic segmentation, localization, and detection)^8^^,^^13^ [Fig. 1(a)]. Simplifying the annotation process reduces both the annotation time and the inter-expert variability. It is a promising avenue in cases where the annotation task proves to be tedious and requires the involvement of trained experts.

In some cases, the available datasets do not provide any or only a very limited number of annotated images, making the training of a deep neural network inconceivable. One promising approach to increase the number of annotated images within a larger dataset in an efficient way is active learning^16^ [Fig. 1(b)]. In an active learning context, the training dataset is iteratively created by asking an expert to label the most informative samples, i.e., those expected to bring the most improvement to the model’s performance, using a measure of prediction uncertainty^17^^,^^18^ or sample diversity.^19^^,^^20^ However, when the annotation cost (i.e., time to produce an annotation) of each sample is not constant across the dataset, it should be considered in the design of the active learning model to avoid increasing the total annotation cost while reducing the number of annotations.^14^^,^^21^ We have shown how a trade-off between annotation cost and model performance can be achieved in a simple task, which could be extended to neurophotonics data.^14^

In neurophotonics datasets, researchers often face the challenge of defining a precise ground truth for a specific task (e.g., identifying object borders for a segmentation task). In this context, large intra- and inter-expert variability can be observed.^13^ Crowdsourcing is a strategy used to gain access to a large pool of annotations from the community. Crowdsourcing helps alleviate the challenge of annotating large amounts of data by spreading the task across many people, also enabling the collection of multiple annotations for each sample. This is initially used to help aggregate the annotations from non-expert users, but it also grants access to a measure of the confidence on the obtained annotations. Such a measure could be leveraged to consider the commonly encountered annotation variability of biological structures in neurophotonics datasets.

Self-supervised learning^22^^,^^23^ (SSL) is a promising avenue for addressing annotated data scarcity when large datasets are available but the accessibility of ground truth annotations is limited. SSL is a two-steps paradigm where (1) the general representation of the domain is learned using a pretext task that does not require labeled data, and (2) the downstream task is learned using the fraction of the dataset that is labeled.^24^ The use of SSL for neurophotonics can be limited by the identification of reliable pretext tasks that are well adapted for microscopy images. Common pretext tasks for images include context prediction,^22^^,^^25^ jigsaw puzzles,^26^ rotation prediction,^27^ and colorization;^28^ all of which are not directly applicable to microscopy images. The context prediction and the jigsaw puzzle tasks could have more than one possible answer (particularly for images where structures are far apart), rotations are not defined in the plane of the image, and pseudo-colors are arbitrarily defined from photon-counts. Instance discrimination,^29^ geometric self-distillation,^30^ classification of image parameters (e.g., scale^31^), and image prediction^32^^,^^33^ are all pretext tasks that are applicable to microscopy images, do not require semantic labels, and yet still enable the model to learn generalizable representations of the data. Image prediction can be used as a pretext task to learn denoising in temporal imaging data, improving signal-to-noise ratio in calcium^34^^,^^35^ and voltage^36^ imaging without the need for ground-truth denoised images, which are difficult to obtain. These methods take advantage of the spatial relationship between consecutive frames to learn to generate images with reduced noise. Such denoising approaches can be applied in real-time during imaging, proving particularly useful in photon-limited contexts, such as two-photon microscopy.^37^

### 2.2 Data Scarcity

The expanding range of microscopes and imaging systems that are routinely applied to neuroscience research questions unlocks new insights into complex biological processes. The gained flexibility in the choice of devices and acquisition parameters to characterize a given biological structure can lead to an increased variability in the properties of the generated datasets for a very similar research question. Images from the same structure acquired by two different groups or even at different time points on the same device can belong to different data distributions. Notably, models trained on a given type of images (dimension, resolution, and modality) will not necessarily be directly applicable to a new dataset.^13^ While it can be obvious when addressing completely different modalities, it becomes problematic when differences between the image datasets are barely perceptible even for a trained expert [Fig. 1(c)].

A model proven to be effective for the segmentation of F-actin nanostructures in STimulated Emission Depletion (STED) images of fixed hippocampal neurons^8^ was unsuccessful in segmenting the same structures on new images acquired a few years later on the same device. To avoid annotating a new dataset to retrain a deep neural network from scratch, we explored two alternative approaches: transfer learning (fine-tuning the original segmentation network) and synthetic data generation using a conditional generative adversarial network (cGAN) for domain adaptation.^15^ cGANs create synthetic images from one domain based on input images from another domain.^38^^,^^39^ It allows adaptation of the image features from a new distribution to match those of the original distribution [Fig. 1(c)]. Both methods (transfer learning and training on domain-adapted synthetic data) improved the segmentation accuracy on the new dataset over the original segmentation network. Synthetic data may also be generated using biologically and optically accurate simulations without the use of neural networks.^40^^,^^41^ This proves useful in particularly data-hungry learning methods, such as reinforcement learning, where acquiring the required amount of imaging data for training is unreasonable.^42^

**Fig. 2:**
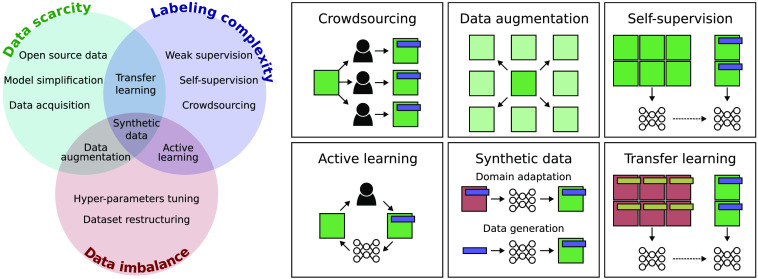
(Left) Solutions to address common data-driven challenges in neurophotonics: data scarcity, label scarcity, and data imbalance. (Right) Green and red boxes represent images from different domains, and blue and yellow rectangles represent annotations for different tasks.

The idea of using knowledge obtained from one dataset before learning a task on a second dataset was proven effective for many applications and is a well-established method for addressing data scarcity.^43^^,^^44^ When training a deep neural network using transfer learning, we rely on a large labeled dataset to learn the initial weights of the model. This model can be fine-tuned to the new domain associated with a smaller dataset (Fig. 2). Using transfer learning reduces the training cost since the pretraining step is performed on very large open-source datasets. However, transfer learning might offer little benefit in neurophotonics since the features defining the structures in microscopy images differ significantly from the features in the common large datasets composed of natural images.^44^ Encouraging the shift toward open-source data will allow building huge field-specific community datasets from which general representations can be learned, similarly to commonly used computer vision datasets.

### 2.3 Data Imbalance

A dataset where the number of training examples is not constant across classes is called imbalanced. Data imbalance is a prevalent challenge across bio-imaging applications, because elements of interest tend to correlate with fewer occurrences.^45^ In neurophotonics, segmentation tasks often meet the data imbalance problem since the number of pixels to which a class is assigned can be far inferior to either the number of pixels from a different class (e.g., cell bodies versus neuritis^46^ and active versus inactive neurons in two-photon calcium imaging^47^) or the number of unlabeled pixels (background or non-studied structures). If this data imbalance is not addressed, models can become overconfident for the more prevalent classes and avoid learning the distinctive features of less common classes.

Data augmentation in computer vision is the process of applying geometric transformations (e.g., rotations, scaling, flips, crops, and skewing), color transformations (e.g., brightness, contrast, and saturation), or appearance transformations (e.g., Gaussian filtering, Sobel filtering, and noise addition) to increase the number of different training examples from a given number of data samples.^48^ Data augmentation can alleviate the consequences of data imbalance by augmenting the images of rare classes until their number matches the most common class. However, not all types of transformations designed for natural images can be applied to microscopy images. For example, scaling images during training could prevent a model from learning size features that can be useful for analyzing images acquired at a constant magnification.^49^ Colors also hold a different meaning in fluorescence microscopy than for natural images and this meaning must be preserved through color transformations.^50^ The application of filters can decrease the resolution of the images, and nanoscale elements can be lost through the blurring effects, affecting the possibility for the model to recognize nanoscale features.^51^ Careful considerations must be taken for what types of transformations can be applied to the images without altering the significance of their assigned labels.

## 3 Conclusion

In the field of neurophotonics, challenges associated with data and label scarcity can be exacerbated by the complexity of the image acquisition, the requirement for expert knowledge for annotations, and the experimental variability. We covered a few possible methods for tackling data and label scarcity through concrete challenges we have encountered. To democratize machine learning-based quantitative bioimaging, the development of approaches that are accessible, reproducible, documented, and broadly available to the community will be essential. Their deployment will be coupled with strategies to improve the efficiency of the training process of deep learning models, both in terms of required data and annotations.
